# Isotope Fractionation of Toluene: A Perspective to Characterise Microbial *In Situ* Degradation

**DOI:** 10.1100/tsw.2002.210

**Published:** 2002-05-08

**Authors:** H.H. Richnow, A. Vieth, M. Kastner, M. Gehre, R.U. Meckenstock

**Affiliations:** UFZ Centre for Environmental Research Leipzig Halle Ltd., Permoserstr. 15, 04318 Leipzig, Germany; Eberhard-Karls University of Tübingen, Center for Applied Geoscience, Sigwartstr. 10, 72076 Tübingen, Germany

**Keywords:** contaminated aquifers, natural attenuation

## Abstract

A concept to assess *in situ* biodegradation of organic contaminants in aquifers is presented. The alteration of the carbon isotope composition of contaminants along the groundwater flow path indicates microbial degradation processes and can be used as an indicator for *in situ* biodegradation. The Rayleigh equation was applied to calculate the percentage of the *in situ* biodegradation (B[%]) using the change in the isotopic composition of contaminants (R_t_/R_0_) along the ground water flow path and a kinetic carbon isotope fractionation factor (*α*C) derived from defined biodegradation experiments in the laboratory. When the groundwater hydrology is known and a representative source concentration (C_0_) for a groundwater flow path can be determined, the extent of *in situ* biodegradation can be quantified.

## INTRODUCTION

*In situ* biodegradation is a major sink of organic contaminants in the aquifer. Thus, *in situ* biodegradation is an important parameter in groundwater monitoring to evaluate *in situ* bioremediation approaches. Today, *in situ* biodegradation is assessed by monitoring the decrease of the concentration of contaminants in time series. However, a decrease of contaminant concentration in a plume could have many causes, such as dilution, sorption, or biological degradation, but only the latter leads to a sustainable contaminant reduction.

Moreover, in the context of economically reasonable remediation strategies, monitored natural attenuation has gained importance. When considering natural attenuation as a reliable remediation strategy, the assessment of *in situ* degradation is the key factor to demonstrate the feasibility of this concept. Thus, innovative approaches have to be developed to monitor processes governing the fate of contaminants in the aquifer.

Biodegradation of pollutants such as aromatic hydrocarbons and chlorinated ethenes leads to an enrichment of $^{13}C$ and $^{2}H$ in the residual fraction[1,2,3]. Dilution and volatilisation processes[4], as well as sorption, are unlikely to induce significant fractionation under field conditions. To characterise *in situ* biodegradation, a concept based on the isotope fractionation of organic contaminants during biodegradation had been developed[1,2,3,4,5] and was tested in several field studies. For this report, we selected three field sites of different sizes and origins of contaminants to demonstrate that the *in situ* biodegradation of toluene can be monitored downstream of the source area by applying the isotope fractionation concept.

## EXPERIMENTAL METHODS/PROCEDURES

### Zeitz

The aquifer is located on the site of a former hydrogenation plant in Zeitz (Saxonia-Anhalt, Germany) and represents a massive contamination resulting from benzene production. The site is characterised by two aquifers, which are separated by silt and clay of Tertiary age and parts of a lignite seam. The upper aquifer is a 5–10 m layer of sandy and clayey deposits of predominantly Pleistocene age. The groundwater table is situated 8 to 10 m below surface. The lower aquifer is composed of sand and gravel of Tertiary age. Both aquifers are partly connected hydraulically. The general groundwater flow direction is to the north. Both aquifers are virtually free of dissolved oxygen (data not shown) suggesting that only anaerobic microbial processes may be involved in the degradation of pollutants in contaminated parts. The prevailing contaminants of the upper aquifer are benzene, toluene, ethylbenzene, and xylene (BTEX), dominated by high benzene concentrations up to 500 mg $l^{-1}$, followed by toluene (50 mg $l^{-1}$), ethylbenzene (1.5 mg $l^{-1}$), and 2.5 mg $l^{-1}$ xylene, respectively. The BTEX concentrations in the aquifer of Tertiary age are considerably lower (100 mg $l^{-1}$), and benzene is again the dominant pollutant.

### Hamburg

The aquifer is located in an industrial area near the city of Hamburg, Germany, and represents a tar oil contamination. The contamination dated back more than 30 years and was caused by leaking storage tanks containing an aromatic oil rich in naphthalene. The groundwater flux generated a contamination plume more than 800 m long. In the contaminated area of the aquifer anoxic conditions are prevailing. The Pleistocene aquifer is composed of Quaternary sands and gravel with a thickness ranging between 7 and 15 m overlying erratic marls (Drenthe I). Large parts of the aquifer were covered by erratic marls (Drente II) leading to confined groundwater conditions with a minor infiltration of surface water along the contamination plume. The groundwater table was 3–10 m below the surface.

### Ingolstadt

The aquifer is located at a motorway junction near the city of Ingolstadt, Germany, and represents a small fuel contamination. The aquifer was contaminated with gasoline as a result of a car accident with a tank lorry. Several cubic m of gasoline, mainly BTEX, contaminated a shallow sandy aquifer and generated a plume of about 25 m in length. The naturally oxic sandy aquifer changed to anoxic conditions in the area of the plume (data not shown).

## METHODS

Groundwater samples were taken and extracted with *n*-pentan. The carbon isotopic composition of the contaminants was measured with an isotope ratio monitoring–gas chromatography–mass spectrometry system (IRM-GC-MS). The IRM-GC-MS system consists of a GC unit connected to a Finnigan MAT combustion device with a water removal assembly coupled to a Finnigan MAT 252 mass spectrometer. All carbon isotope ratios are reported in the delta notation as $\delta^{13}C$ values (‰) in relation to Pee Dee belemnite (PDB).

## CALCULATIONS

The Rayleigh equation (Eq. 1) was applied to describe the fractionation process, where $R_t$ gives the isotope ratio $^{13}C/^{12}C$ at time $t$, $R_0$ gives the isotope ratio $^{13}C/^{12}C$ at time 0, and $f$ is the fraction of substrate remaining at time $t$ ($f = C_t/C_0$). The kinetic isotope fractionation factor ($\alpha C$) gives the relation between concentration and isotopic composition of a compound. When ln $R_t/R_0$ is plotted over ln $C_t/C_0$, the slope of the linear regression curve expresses $\alpha C$ as ($1\alpha/C - 1$) (Eq. 2). Based on

$$\frac{R_t}{R_0} = \left(\frac{C_t}{C_0}\right)^{\left(\frac{1}{aC}-1\right)} \tag{1}$$

$$\ln\left(\frac{R_t}{R_0}\right) = \left(\frac{1}{aC}-1\right)\times \ln\left(\frac{C_t}{C_0}\right) \tag{2}$$

$$\frac{C_t}{C_0} = \left(\frac{R_t}{R_0}\right)^{\left(\frac{1}{\frac{1}{aC}-1}\right)} \tag{3}$$

$$B[\%] = \left[1-\left(\frac{R_t}{R_0}\right)^{\left(\frac{1}{\frac{1}{aC}-1}\right)}\right]\times 100 \tag{4}$$

$$C_t = C_0 \times \left(\frac{R_t}{R_0}\right)^{\left(\frac{1}{\frac{1}{aC}-1}\right)} \tag{5}$$

**FIGURE 1.** Equations used for calculations.

the measured isotope values, the percentage of biodegradation can be calculated (Eqs. 3 and 4). The "percentage of biodegradation" is an index to describe the extent of biodegradation. Here the variation of isotopic ratios measured in the field ($R_t/R_0$) and a compound-specific fractionation factor ($\alpha C$) from a biodegradation experiment with pure culture are used to calculate the extent of biodegradation.

The remaining substrate fraction ($C_t$) along a groundwater flow path can be calculated using the isotope fractionation factor ($\alpha C$), the isotope ratios measured in the field ($R_t/R_0$) and the concentration in the area of the source of contaminants ($C_0$) (Eqs. 3 and 5). The concentration ($C_0$) must be representative for the input concentration of a contaminant for a given groundwater flow path.

The carbon isotope fractionation factor ($\alpha C$ = 1.0017) used to calculate the *in situ* biodegradation of toluene was obtained from anaerobic biodegradation experiments with pure cultures[1].

## RESULTS AND DISCUSSION

### Zeitz

Concentrations and carbon isotopic signatures of toluene were measured in 16 groundwater samples from the upper aquifer. In these samples the toluene concentrations were in a range between 50 mg $l^{-1}$ and 5 µg $l^{-1}$. In the centre of the plume the carbon isotope ratios were around –27‰. However, further downstream the isotopic signature of toluene was enriched in $^{13}C$, leading to isotope ratios of –23‰. Within this groundwater flow path the carbon isotopic enrichment of up to 4 δ-units indicates *in situ* biodegradation (Fig. 2). The percentage of biodegradation was calculated for a selected groundwater flow path according to equation 4 (Fig. 3). The isotopic ratio of the

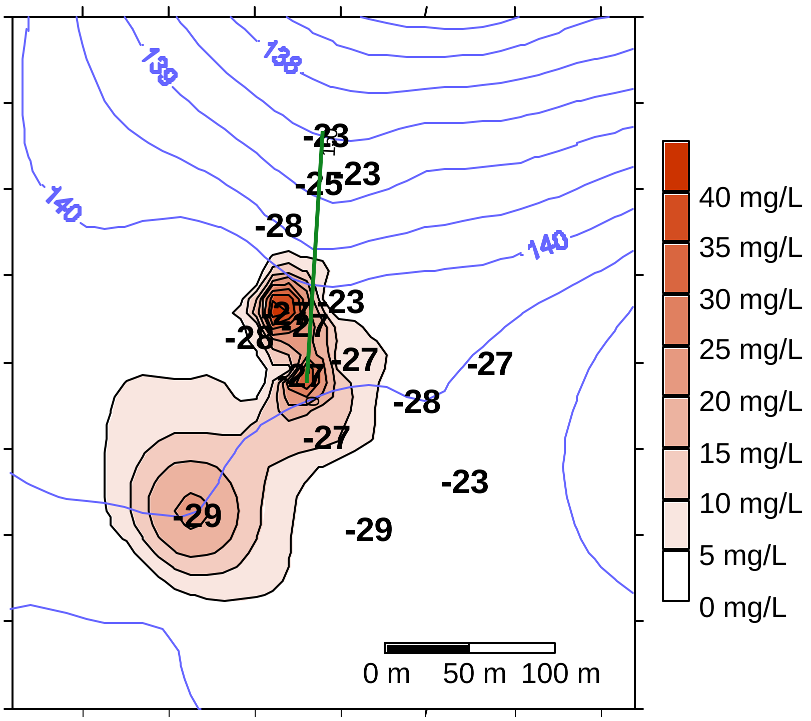

**FIGURE 2.** Concentration and isotopic composition of toluene in the upper aquifer in Zeitz, Germany. The blue lines indicate the depth of the groundwater table above the sea level (m). Black figures show the location of monitoring wells and the isotopic composition of toluene. The green line shows the position of the monitoring profile depicted in Fig. 3.

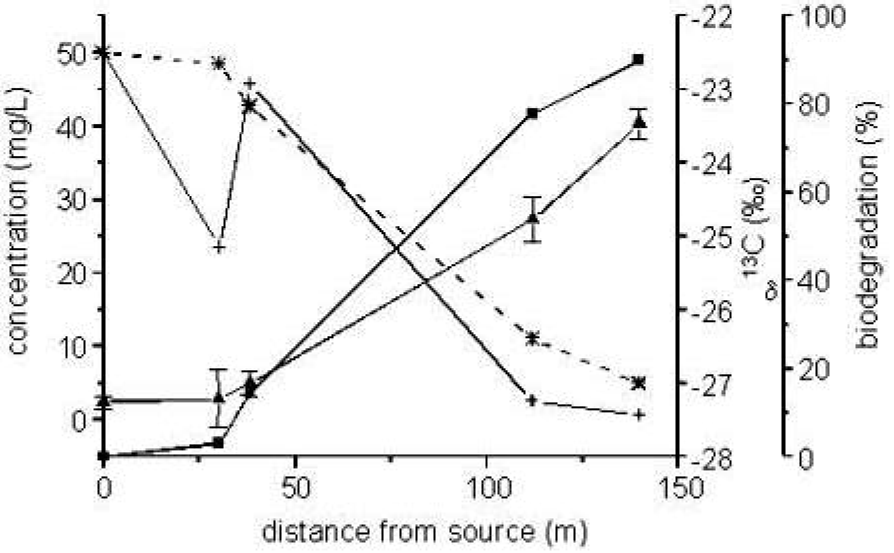

**FIGURE 3.** Concentration (crosses) and isotope composition (triangles) of toluene along the monitoring profile depicted in Fig. 2. The percentage of biodegradation (squares) and the residual substrate fraction (stars) were calculated with Eqs. 4 and 5 using an isotope fractionation factor characteristic for anaerobic biodegradation of toluene.

toluene source in this area of the plume was –27.2‰ assuming that the highest local concentration of toluene (50 mg $l^{-1}$) represents a less degraded toluene fraction. In the second to fifth monitoring wells, the isotopic signature of toluene was enriched in $^{13}C$ up to –24.4‰, indicating intensive *in situ* biodegradation. The shift in the isotope ratios was employed to calculate the percentage of biodegradation ($B$[%], Eq. 4) using the fractionation factor for anaerobic biodegradation of toluene ($\alpha C$ = 1.0017). The index "percentage of biodegradation" clearly increases along the water flow path describing the zones of intensive biodegradation.

The concentration of the remaining toluene fraction can be calculated with Eq. 5 (Fig. 3). Therefore, it was assumed that the isotope ratio of –27.2‰ and concentration of 50 mg·$l^{-1}$ represented the local source of toluene relevant for the water flow path. The calculated residual toluene concentrations were similar to the measured toluene concentrations downstream the groundwater flow path, although clear differences between measured and calculated concentrations were obvious at the second groundwater well. Overall, the concentration gradient can be explained by *in situ* biodegradation, suggesting that microbial processes govern the fate of toluene in this particular field site.

## Hamburg

Along the 800 m profile downstream of the contamination source, toluene showed a significant concentration gradient decreasing from 160 µg $l^{-1}$ to below the detection limit of 0.5 µg $l^{-1}$. With decreasing concentrations, the $^{13}C/^{12}C$ isotope ratio in the residual toluene fraction was enriched up to 7.2 ± 1‰ between the first and fifth wells (Fig. 4). Using the carbon isotope fractionation factor for anaerobic toluene degradation ($\alpha C$ = 1.0017) the percentage of biodegradation was calculated. The isotopic composition at the first well was used as the initial isotopic composition of a theoretical source ($R_0$) to calculate the extent of the biodegradation (Fig. 4). However, applying the isotope value $\delta^{13}C$ at the third well, the remaining toluene fraction had been biodegraded by about 54%. Further downstream from this well, the calculations indicate that the percentage of biodegradation increased to more than 99% at the fifth well.

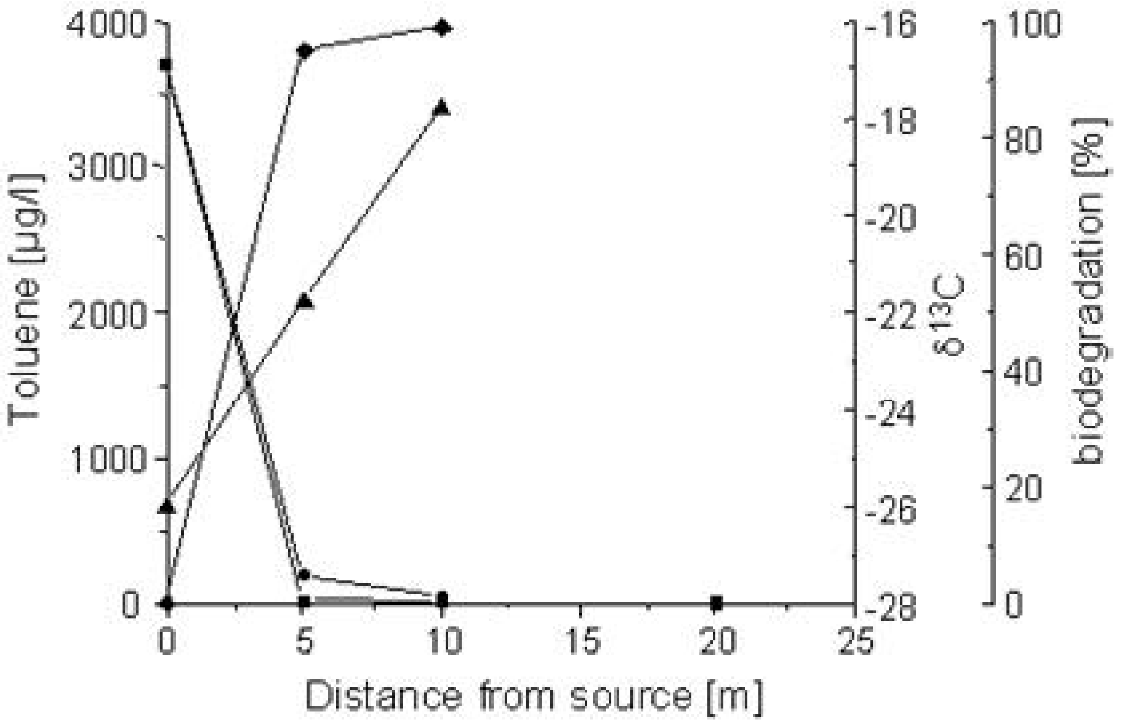

**FIGURE 4.** Concentrations (squares) and $^{13}C/^{12}C$ isotope ratios (triangles) of toluene along the monitoring profile (A – E) of a contaminated aquifer (Hamburg, Germany). The percentage of biodegradation (circles) and theoretical residual toluene fraction (diamonds) are calculated with Eqs. 4 and 5 using a carbon isotope fractionation factor for anaerobic biodegradation (adapted from [6]).

The extent of biodegradation of the residual substrate fraction can be quantified assuming that all monitoring wells were located directly downstream of the source of contamination. Based on this assumption, the isotopic compositions and concentrations monitored in the first well could be employed as $R_0$ and $C_0$ to calculate the absolute amount of biodegraded toluene in the second to fifth wells. The residual toluene concentrations calculated showed a slow decrease for the second and third wells, only roughly matching the residual concentrations measured (Fig. 4). However, further downstream the calculated concentrations decreased strongly, reflecting the measured toluene concentrations in the wells.

## Ingolstadt

Concentration and isotopic composition of toluene was determined along a profile of 25 m downstream the water flow path (Fig. 5). The toluene concentrations showed a steep gradient between 0 and 5 m and were still considerably high until 10 m distance from the first well. The corresponding isotope ratios $\delta^{13}C$ of the residual toluene increased significantly, implying relevant biodegradation in the aquifer. The percentage of biodegradation (*B*[%]), calculated as mentioned above, showed that within a distance of 10 m from the first monitoring well of the profile the toluene concentration was reduced to more than 99% by biodegradation (Fig. 5). The concentration of the residual toluene fraction was calculated with the assumption that all wells were located directly downstream of the contamination source and the first well was a representative source ($C_0$). The calculated residual toluene concentrations show a strong decrease in the wells at 5 and 10 m distance from the source, matching the residual concentrations observed in the field (Fig. 5). The calculated residual concen-

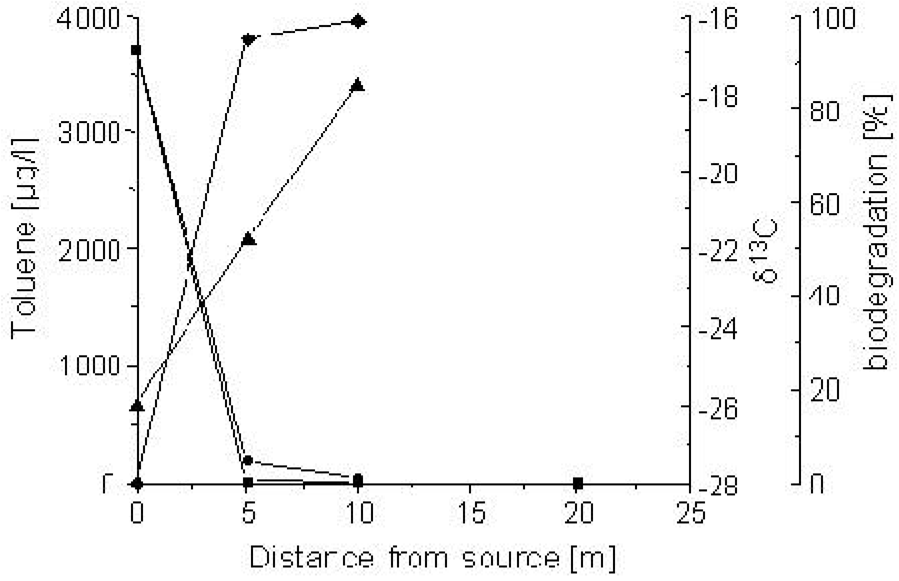

**FIGURE 5.** Toluene concentration (squares) and carbon isotope composition (triangles) along a monitoring profile of a contaminated site near the city of Ingolstadt, Germany. The percentage of biodegradation (diamonds) and theoretical toluene concentrations (circles) are calculated with Eqs. 4 and 5 using a carbon isotope fractionation factor for anaerobic biodegradation. (adapted from [7])

trations were slightly higher than the measured concentrations, which might be a consequence of dilution or adsorption as additional sinks of toluene.

## CONCLUSIONS

This paper shows the various aspects of using isotope fractionation to characterise *in situ* biodegradation. Isotope fractionation may be well used to evaluate the biodegradation in contaminated aquifers. The percentage of biodegradation shows zones of biodegradation and serves as a semiquantitative index. This index is independent of the groundwater flow and dilution or adsorption because it is solely based on isotope values, which are altered during *in situ* biodegradation. Other sinks may also have contributed to the reduction of the contaminant concentrations, but they did not affect the isotope ratios and thus had no effect on the results[1]. This index may be well used as an indicator of the intensity of biological degradation processes in distinct zones of an aquifer.

The theoretical concentration of the residual substrate fraction was quantified, assuming that (I) all monitoring wells were located directly downstream of the source of contamination, and (II) dilution and adsorption did not affect the concentrations to a major extent. When source concentration ($C_0$) is representative for the groundwater flow path, the concentration of the residual fraction ($C_t$) can be calculated. On the three test sites, the calculated residual concentrations are comparable to the toluene concentrations observed in the field, which indicates that *in situ* biodegradation was the major sink of toluene.

In the context of natural attenuation, the stable isotope chemistry may be well used to characterise *in situ* biodegradation. The concept to quantify *in situ* biodegradation may have a large potential to improve groundwater remediation strategies.

## ACKNOWLEDGMENTS

This paper was presented at the CSIC/ESF workshop Analysis, Toxicity and Biodegradation of Organic Pollutants in Groundwater from Contaminated Land, Landfills and Sediments, Barcelona, Spain, 8–10 November, 2001. The work was supported by the Federal Ministry of Education and Research, Germany (BMBF), grant 02WT0022.
